# Properties of the newly isolated extracellular thermo-alkali-stable laccase from thermophilic actinomycetes, *Thermobifida fusca* and its application in dye intermediates oxidation

**DOI:** 10.1186/2191-0855-3-49

**Published:** 2013-08-28

**Authors:** Cheng-Yu Chen, Yu-Chun Huang, Chien-Mei Wei, Menghsiao Meng, Wen-Hsiung Liu, Chao-Hsun Yang

**Affiliations:** 1 Graduate Institute of Biotechnology, National Chung Hsing University, Taichung 40227, Taiwan; 2 Department of Cosmetic Science, Providence University, Taichung 43301, Taiwan; 3 Department of Biochemical Science & Technology, National Taiwan University, Taipei 10617, Taiwan

**Keywords:** Laccase, Dye intermediate, Thermophilic, *Thermobifida fusca*, LC-MS/MS

## Abstract

Laccases are diphenol oxidases that have numerous applications to biotechnological processes. In this study, the laccase was produced from the thermophilic actinomycetes, *Thermobifida fusca* BCRC 19214. After 36 h of fermentation in a 5-liter fermentor, the culture broth accumulated 4.96 U/ml laccase activity. The laccase was purified 4.64-fold as measured by specific activity from crude culture filtrate by ultrafiltration concentration, Q-Sepharose FF and Sephacryl™ S-200 column chromatography. The overall yield of the purified enzyme was 7.49%. The molecular mass of purified enzyme as estimated by SDS-PAGE and by gel filtration on Sephacryl™ S-200 was found to be 73.3 kDa and 24.7 kDa, respectively, indicating that the laccase from *T. fusca* BCRC 19214 is a trimer. The internal amino acid sequences of the purified laccase, as determined by LC-MS/MS, had high homology with a superoxide dismutase from *T. fusca* YX. Approximately 95% of the original activity remained after treatment at 50°C for 3 h. and approximately 75% of the original activity remained after treatment at pH 10.0 for 24 h. This laccase could oxidize dye intermediates, especially 2,6-dimethylphenylalanine and *p*-aminophenol, to produce coloring. This is the first report on laccase properties from thermophilic actinomycetes. These properties suggest that this newly isolated laccase has potential for specific industrial applications.

## Introduction

Laccases (E.C. 1.10.3.2) are well-known enzymes that were first isolated from the lacquer tree, *Rhus vernicifera*. They have received increasing attention in recent decades due to their ability to oxidize both phenolic and nonphenolic lignin-related compounds and highly recalcitrant environmental pollutants, which makes them very useful for applications related to biotechnological processes (Mukhopadhyay et al., 2013; Couto and Herrera, 2006; Albino et al., 2004). Laccases are members of the multi-copper oxidase family of enzymes that contain four copper atoms in their functional units. Laccases have been isolated from many plants, fungi and bacteria. Most known laccases are of fungal origin, and they participate in a variety of physiological functions, such as stress defense and lignin degradation (Baldrian, 2006; Giardina et al. 2010; Kües and Rühl 2011).

One application for laccase is hair coloring. Oxidation-based hair color, which has dominated the hair color market, consists of dye intermediates and an oxidizing agent (Saito et al., 2012). In a typical hair color product, the dye intermediates are *p*-diamines and *p*-aminophenols, and hydrogen peroxide is used as the oxidant in the dyeing process. After mixing, they form chromatic indo dyes at the time of use. However, side reactions with hair proteins commonly occur simultaneously because of the severe reactions conditions, resulting in hair damage (Saito et al., 2012). The commercial hydrogen peroxide oxidative-type hair dyeing formulations are mutagenic. Some hair dyeing components become strongly mutagenic after oxidation by hydrogen peroxide (Ames et al., 1975). Laccase-based hair dyes are less irritating and easier to handle than current hair dyes, as laccases replaced hydrogen peroxide as the oxidizing agent in the dyeing formula (Couto and Herrera, 2006).

Streptomycetes are widespread soil actinomycetes that play important roles in the decomposition of biopolymers such as lignin, cellulose, hemicellulose, chitin, keratin and pectin (Locci, 1989). Although most laccases are found in mesophilic microorganisms, thermophilic microorganisms are considered to be good sources of thermostable and novel enzymes with potential industrial importance. However, little has been reported on laccase production by thermophilic actinomycetes. To produce enzymes for the development of enzymatic degradation of renewable lignocellulose, we isolated 70 potent extracellular lignocellulolytic enzyme-producing thermophilic actinomycetes from compost soils collected in Taiwan (Yang et al., 2009). Of the 70 strains of thermophilic actinomycetes, strain No. 10–1 had the best laccase activity. According to its biological properties and 16S rDNA similarity, this newly isolated strain was identified as *Thermobifida fusca* and deposited in the Bioresource Collection and Research Center (BCRC, Hsinchu, Taiwan) with stock number BCRC 19214 (Chen et al., 2013). Database mining of the complete genome sequence of *T. fusca* YX, which was accessible in 2007 (Lykidis et al. 2007), did not find genes that encode fungal laccase-like proteins. There are no reports on laccase production and properties by *T. fusca* before.

The coloring reactions are usually carried out at alkaline pH because the hair swells and the penetration of dyes is enhanced. Although many laccases have been isolated, few studies have reported laccases with high activity under neutral or alkaline conditions (Gouka et al., 2001; Heinzkill et al., 1998; Sulistyaningdyah et al., 2004). The purpose of this study was to produce thermo-alkali-stable laccase from thermophilic actinomycetes. The enzyme properties and dye intermediates oxidation application of the laccase were also investigated. The results of this investigation have implications for cosmetics and human health.

## Materials and methods

### Microorganisms

*T. fusca* BCRC 19214, cultivated routinely in CYC medium (Czapek-Dox powder 33 g/L, yeast extract 2 g/L, casamino acids 6 g/L, pH 7.2) at 50°C, was studied in this study. This strain was deposited in the Bioresource Collection and Research Center (BCRC, Hsinchu, Taiwan) with stock number.

### Materials

Sugarcane bagasse, corncob, and pine sawdust were collected from the local market and washed extensively with running water until the residual soluble sugar was removed. Then, they were dried and mixed in a blender. The resulting small pieces, passed through a 100 mesh screen, were collected and used in this study. Czapek-dox powder, yeast extract, casamino acids, and agar were obtained from BD (Sparks, MD, USA). Q-Sepharose FF and Sephacryl™ S-200 were supplied by GE Healthcare (Little Chalfont, UK). The protein assay kit and SDS-PAGE molecular weight standards were obtained from Bio-Rad Laboratories (Hercules, CA, USA). Dye intermediates, inorganic salts and all other chemicals were purchased from Sigma (St. Louis, MO, USA).

### Cultivation in a fermentor

The laccase-producing strain was cultivated in a 5-liter fermentor (Biostat B, B. Braun, Melsungen, Germany). A 500-ml Hinton flask containing 100 ml of bagasse medium was inoculated with the strain cultured at 50°C and shaken (150 rpm) for 48 h; this was used as the seed culture. The 5-liter fermentor was loaded with 3 liters of bagasse medium. Cultivation was performed at 50°C, 1.0 v.v.m. aeration and 300 rpm agitation.

### Enzyme purification

All purification procedures were performed at 4°C in 20 mM phosphate buffer (pH 8.0) unless otherwise stated. After 36 h cultivation of the laccase-producing strain in a 5-liter fermentor, the fermentation broth was centrifuged at 10,000 × *g* for 30 min to remove the cells. The supernatant was concentrated by ultrafiltration (Pellicon XL, Biomax 10 K, Millipore). The concentrated solution was then applied to a Q-Sepharose FF anion-exchange column (1.13 cm × 5 cm) that was pre-equilibrated with phosphate buffer. After washing with the same buffer to remove inactive protein, the enzyme was eluted with a linear gradient of the buffer containing NaCl from 0.0 M to 1.0 M (flow rate: 180 ml/h). Enzyme activity was detected within the range 0.4 M to 0.6 M NaCl. The main active fractions were applied to a Sephacryl™ S-200 column (1.6 × 90 cm) that was previously equilibrated with 20 mM phosphate buffer. Proteins were eluted at a flow rate of 30 ml/h. The eluted enzymatically active fractions were pooled and used as the purified enzyme.

### Polyacrylamide gel electrophoresis

SDS-PAGE was performed using 10% gels to determine the molecular weight of the purified protein and the purity of each purification step. Coomassie Brilliant Blue R-250 was used for protein staining. LMW-SDS Maker (GE Healthcare, Little Chalfont, UK) was used as the standard. Native-10% PAGE was performed according to standard SDS-PAGE procedures, but the gels did not contain SDS. The samples were not heated, and no SDS or β-mercaptoethanol was added. The electrophoresis was performed at 150 V for 1 h at 4°C. After electrophoresis, the native-PAGE was washed in 20 mM phosphate buffer (pH 8.0) at 4°C and 50 rpm for 90 min, with buffer changes every 30 min. The native-PAGE was soaked in 20 mM 2,6-DMP (dissolved in 20 mM phosphate buffer ) and then incubated at 50°C until bands began to appear.

### Internal amino acid sequence of laccase by LC–MS/MS

The laccase internal amino acid sequence was performed by in-gel digestion of the protein and sequencing of the different peptides by liquid chromatography tandem mass spectrometry (LC-MS/MS) using an Applied Biosystems QStar LC-MS/MS spectrometer (Life Technologies Corp., Carlsbad, USA) as described previously (Chen et al., 2013). The analysis was performed at the Biotechnology center, NCHU (National Chung Hsing University). The mass spectrometry information was analyzed using Mascot software (Matrix Science Ltd., London, UK) and the NCBInr database. The peptide mass accuracy was ±0.5 Da for Mascot analysis. The resulting amino acids were matched to the NCBI database.

### Laccase activity

Unless otherwise indicated, the standard laccase activity assay was carried out at 50°C for 15 min, using 20 mM 2,6- dimethoxyphenol (2,6-DMP) as substrate in 20 mM phosphate buffer (pH 8.0). 2,6-DMP oxidation was monitored by the increase in absorbance at 470 nm (ϵ470 = 35645 M^-1^ cm^-1^). One unit was defined as the activity required to oxidize 1 nmol of the substrate per minute under the indicated reaction conditions (Chen et al., 2013).

### Oxidative activities for dye intermediates

**Figure 1:**
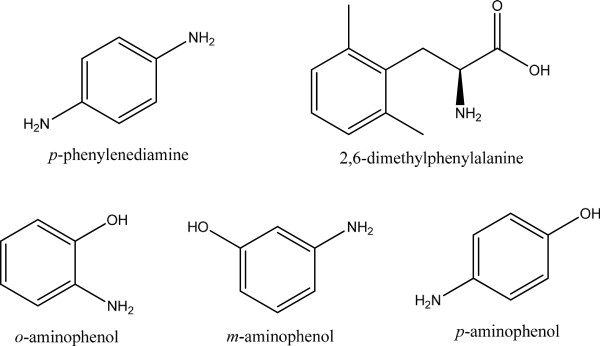
Chemical structure of dye intermediates.

The dye intermediates used in this study are shown in Figure 1. The oxidation activities using *p*-phenylenediamine, 2,6-dimethylphenylalanine, and aminophenols were determined in a DMSO solution (final concentration was 0.5%). Oxidation was spectroscopically monitored in phosphate buffer (pH 8.0). The final concentrations of the substrates and the buffer were 20 mM. The oxidation was monitored at 470 nm.

### Statistical analysis

All analytic measurements were performed at least 3 times. The data are expressed as the mean ± SD.

## Results

### Production of the laccase in fermentor

**Figure 2:**
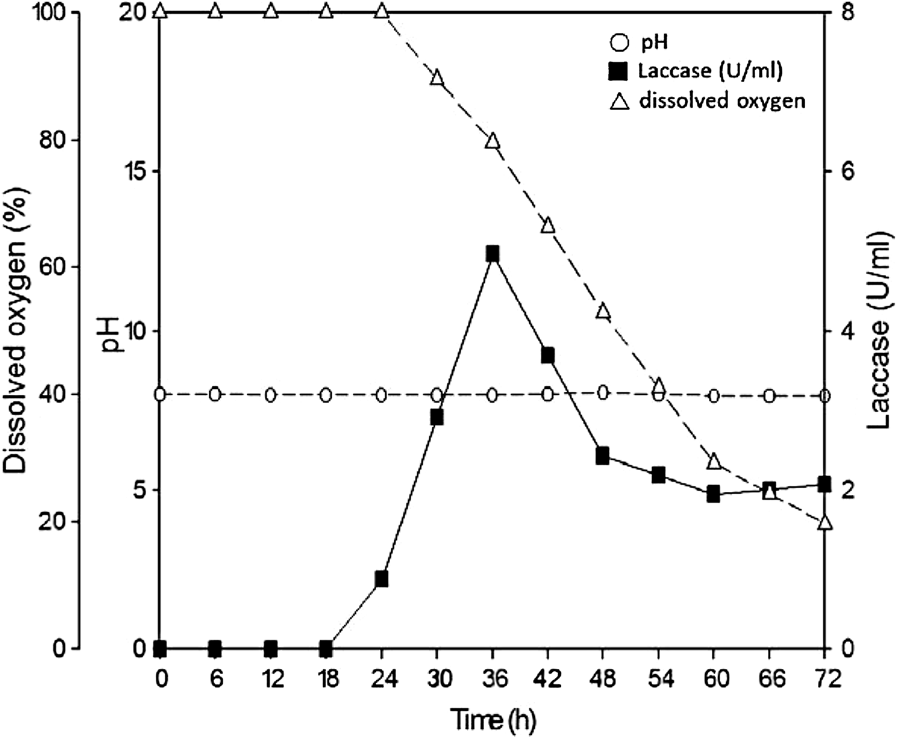
**Time course for production of laccase in a 5-liter fermentor by *T. fusca *BCRC 19214.** The operation conditions were as follows: working volume, 3 liter; inoculum size, 1%; agitation speed, 300 rpm; aeration rate, 1 vvm; temperature, 50°C. Symbols: (■) laccase, (△) dissolved oxygen, (○) pH.

Bagasse, corncob and pine sawdust were used as carbon sources to produce the laccase from *T. fusca* BCRC 19214. The highest extracellular laccase activity was observed when *T. fusca* BCRC 19214 was grown in a mineral medium containing bagasse as the carbon source. Next, the culture conditions for the production of laccase were investigated in a 5-liter fermentor. Typical enzyme levels produced by *T. fusca* BCRC 19214 in a 5-liter fermentor are shown in Figure 2. The rapid consumption of oxygen paralleled an increase in laccase activity during the growth phase of the culture. The appearance of laccase activity in the culture broth became significant after 24 h of cultivation. Maximum laccase activity was approximately 4.96 U/ml in the culture broth after 36 h of cultivation, and the laccase activity decreased after further cultivation time.

### Purification of laccase from *T. fusca* BCRC19214

**Figure 3:**
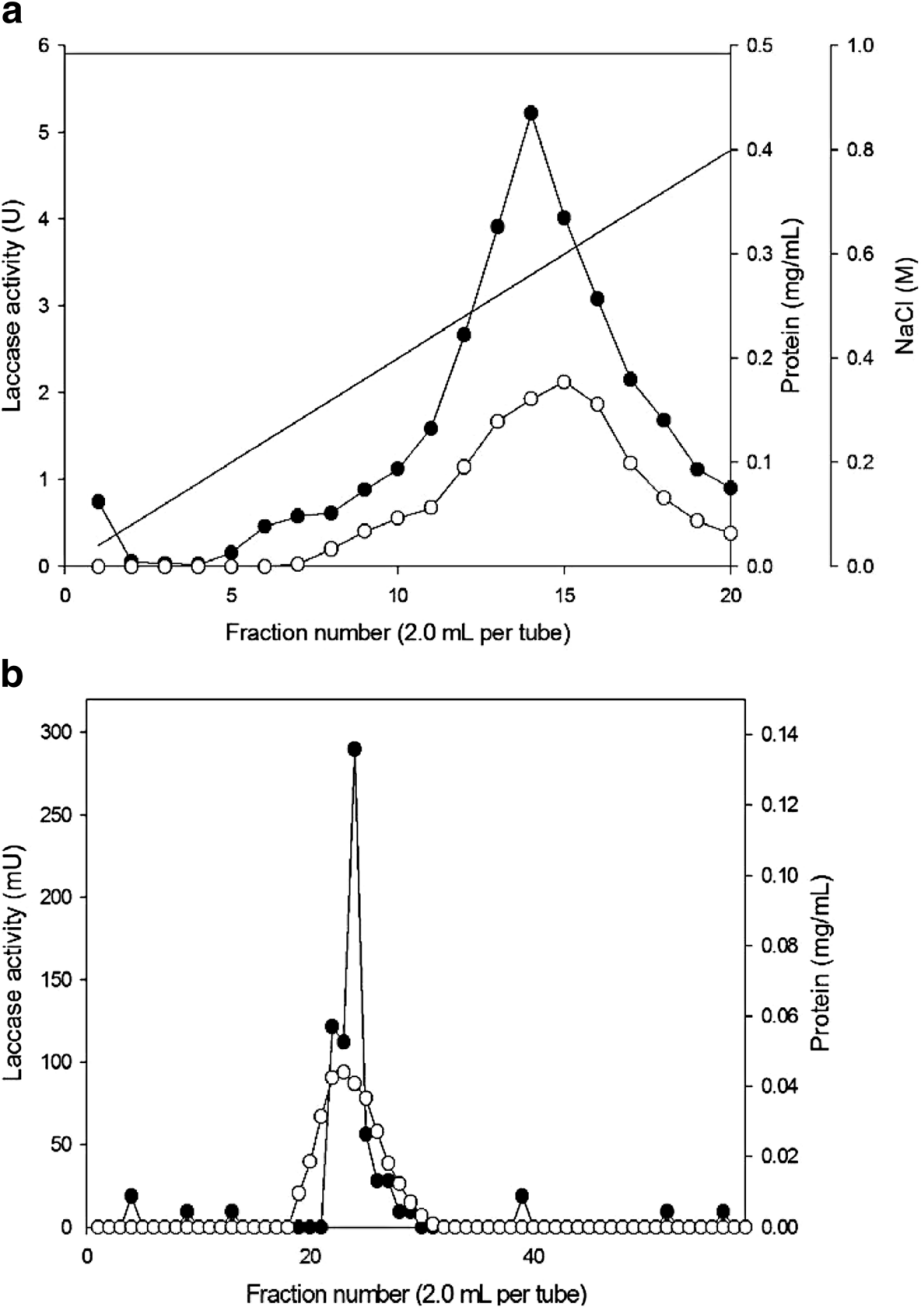
**Purification of laccase by column chromatography. (a)** Q-Sepharose FF chromatography. (●) laccase activity; (○) Protein (mg/ml); (−) NaCl gradient. Column: 1.13 × 5 cm; flow rate: 180 ml/h. **(b)** Sephacryl™ S-200 chromatography. (●) laccase activity; (○) Protein (mg/ml); (−) NaCl gradient. Column: 1.6 × 90 cm; flow rate: 30 ml/h.

The purification of laccase was performed as described in the Materials and Methods section. Gel-filtration chromatography of Q-Sepharose FF is shown in Figure 3a. The major esterase activity fraction was eluted and applied to a DEAE-Sepharose CL-6B column (Figure 3b). The enzymatically active fractions were eluted, pooled and used as the purified enzyme. The results of the total purification are summarized in Table 1. The purified enzyme obtained exhibited 7.49% of the total initial activity and there was a 4.64-fold increase in its specific activity compared with the crude culture filtrate.

**Table 1:** **Summary of the purification of laccase from *T. fusca *BCRC 19214**

**Step**	**Volume (mL)**	**Total protein (mg)**	**Total activity (U)**	**Specific activity (U/mg)**	**Purification (fold)**	**Yield (%)**
Crude culture filtrate	760.00	12.31	49.04	3.98	1.00	100.00
Ultrafiltration	20.00	7.94	39.09	11.27	2.83	79.71
Q-Sepharose FF	16.00	2.36	30.75	13.05	3.28	62.71
Sephacryl™ S-200	6.00	0.20	3.68	18.50	4.64	7.49

### Properties of laccase from *T. fusca* BCRC19214

**Figure 4:**
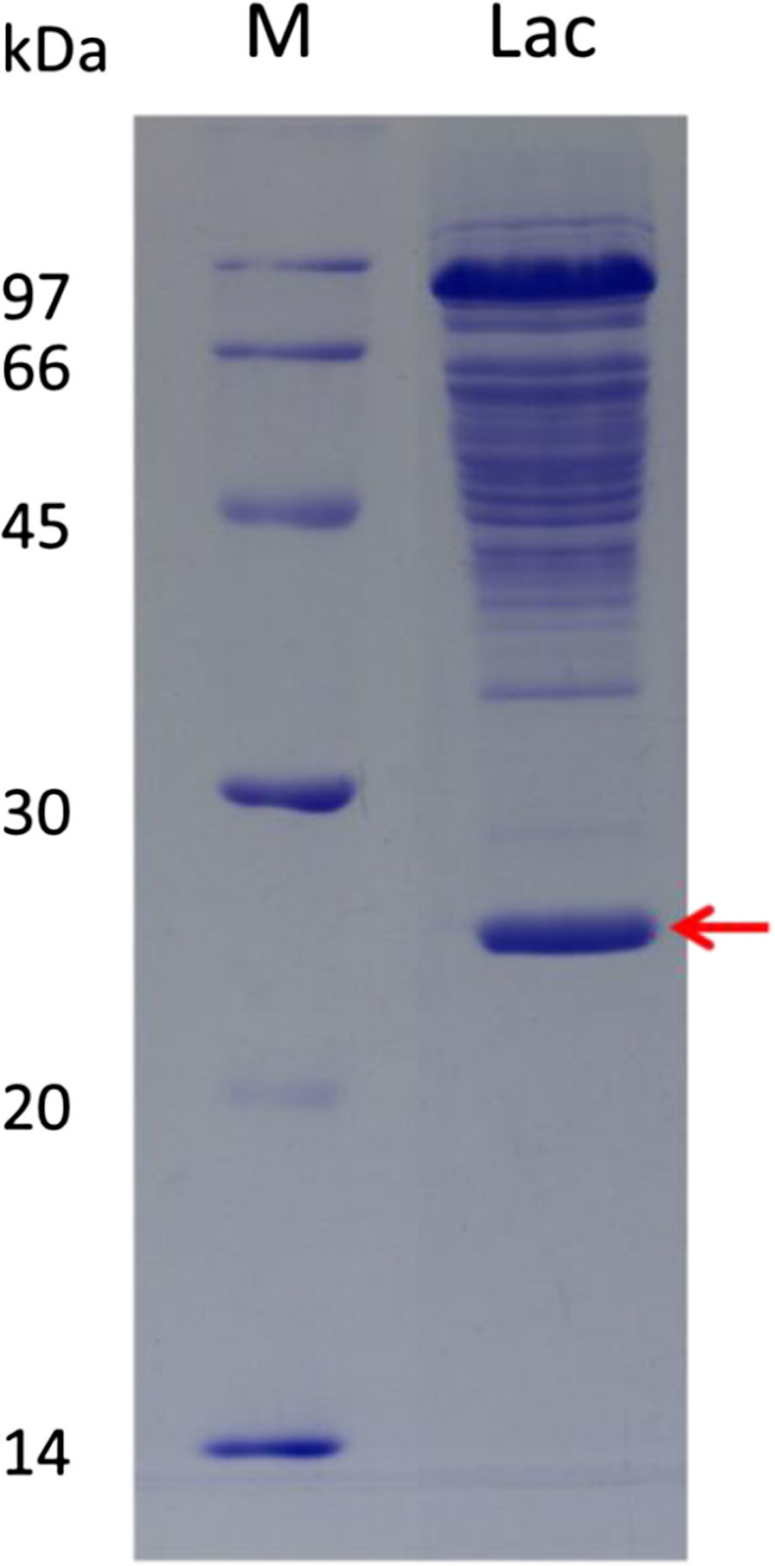
**SDS-PAGE of the purified laccase from *T. fusca *BCRC 19214.** Lane M, Molecular weight marker; Lane Lac, Purified laccase.

As shown in Figure 4, the purified enzyme showed an apparent single protein band on SDS-PAGE (10% gel). The subunit of the single protein band was estimated to be 24.7 kDa from its mobility relative to those of standard proteins on SDS-PAGE. The molecular mass of the purified enzyme was estimated to be 73.3 kDa by Sephacryl™ S-200 gel filtration, indicating that the purified enzyme is a trimer.

**Figure 5:**
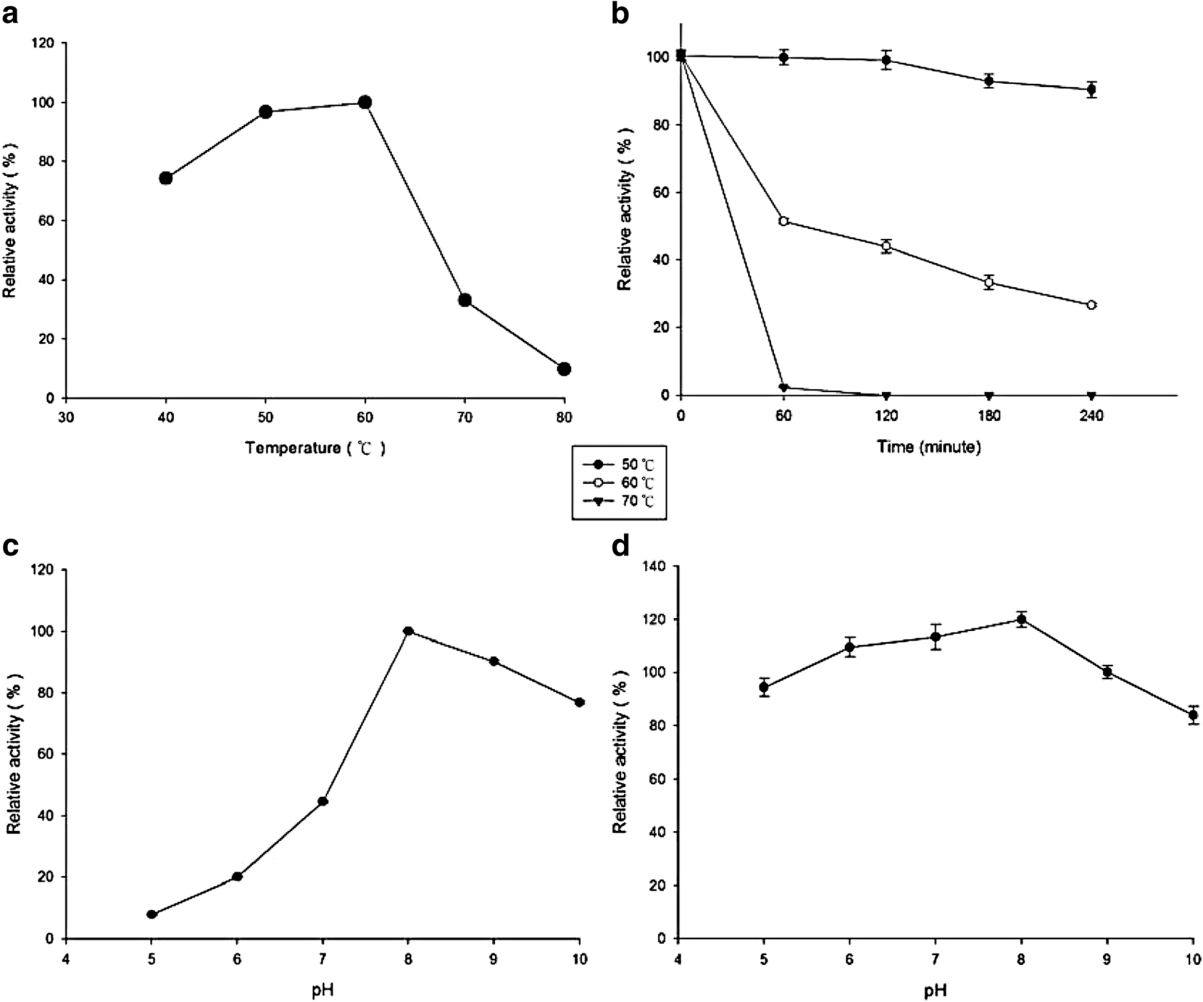
**Effects of temperature and pH on stability and activity of purified laccase from *T. fusca *BCRC 19214. (a)**, the activity was measured at various temperatures 40-80°C in pH 8.0 for 15 min. **(b)**, the residual activity, measured with 2,6-DMP at 50°C, was determined after the enzyme had been incubated at 50-70°C in pH 8.0 for 0–240 min. **(c)**, the activity was measured in various buffer solutions, pH 5.0-10.0, at 50°C. Glycine-NaOH buffer (20 mM, pH 10.0), Tris–HCl buffer (20 mM, pH 8.0-9.0), phosphate buffer (20 mM, pH 6.0-7.0), and citric acid-citrate buffer (20 mM, pH 5.0) were used. **(d)**, the residual activity, measured at 50°C, was determined after the enzyme had been incubated in various buffer solutions, pH 6–10, at 4°C for 24 h. These data are representative of three replicate experiments.

Activity assays were performed using 2,6-DMP as substrate to identify the pH and temperature properties. The optimal pH and temperature of the purified enzyme were 8.0 and 60°C, respectively (Figure 5). The purified enzyme was stable over a pH range of 5.0-10.0 at 4°C for 24 h. Approximately 95% of the original activity remained after treatment at 50°C for 3 h. Approximately 75% of the original activity remained at pH 10.0 for 24 h.

Among the various metal salts and chemical reagents tested, it was found that the purified enzyme activity was partially inhibited by 1 mM Hg^2+^ (Table 2). The presence of β-mercaptoethanol and EDTA significantly reduced the enzyme activity. The enzyme activity partially inhibited by sodium dodecyl sulfate (SDS) and sodium azide (Table 3). The effects of organic solvent on the activity of purified laccase were given in Table 4. With 20% water miscible solvent like methanol, ethanol, isopropanol, aceton, and dimethyl sufoxide (DMSO) in the reaction system, the activity dropped to 20-74%. Acetonitrile and dimethylformamide (DMF) increased the activities to 110-118%.

**Table 2:** **Effect of metal salts on the activity of laccase from *T. fusca *BCRC 19214**

**Metal salt (1 mM)**	Relative activity* (%)
None	100
ZnCl_2_	97 ± 4
HgCl_2_	86 ± 6
CaCl_2_	102 ± 2
FeCl_2_	97 ± 4
MnCl_2_	96 ± 3
MgCl_2_	98 ± 4
NaCl	98 ± 6
KCl	98 ± 3
CoSO_4_	97 ± 4
CuSO_4_	102 ± 5

* The relative activity was measured at 50°C for 15 min, using 20 mM 4- dimethoxybenzylalcohol (veratryl alcohol) as the substrate in 20 mM phosphate buffer (pH 8.0).

**Table 3:** **Effect of inhibitors on the activity of laccase from *T. fusca *BCRC 19214**

**Inhibitor**	**Concentration (mM)**	Relative activity* (%)
None	-	100
SDS	0.1	94 ± 3
	1	73 ± 3
	10	63 ± 6
sodium azide	0.1	87 ± 2
	1	84 ± 4
	10	80 ± 3
⋅-mercaptoethanol	0.1	86 ± 3
	1	4 ± 2
	10	0 ± 1
EDTA	0.1	24 ± 2
	1	23 ± 2
	10	5 ± 1

* The relative activity was measured at 50°C for 15 min, using 20 mM 2,6- dimethoxyphenol (2,6-DMP) as the substrate in 20 mM phosphate buffer (pH 8.0). SDS, Sodium dodecyl sulfate; EDTA, Ethylenediaminetetraacetic acid.

**Table 4:** **Effect of organic solvents on the activity of laccase from *T. fusca *BCRC 19214**

**Organic compounds**	**Concentration (%, v/v)**	Relative activity* (%)
None	-	100
Methanol	10	59 ± 3
	20	34 ± 3
Ethanol	10	66 ± 3
	20	40 ± 4
Isopropanol	10	61 ± 2
	20	36 ± 4
Acetone	10	50 ± 2
	20	20 ± 1
Dimethyl sulfoxide	10	101 ± 1
(DMSO)	20	74 ± 4
Acetonitrile	10	160 ± 6
	20	118 ± 5
Dimethylformamide	10	120 ± 4
(DMF)	20	110 ± 5

* The relative activity was measured at 50°C for 15 min, using 20 mM 2,6- dimethoxyphenol (2,6-DMP) as the substrate in 20 mM phosphate buffer (pH 8.0).

### Internal amino acid sequence of the laccase by LC–MS/MS

The internal sequences of the purified laccase were determined by digestion with trypsin and sequence analysis using LC–MS/MS. Three sequences (GANDALEQLAEAR, AHFSAAATGIQGSGWAILAWDILGQR, and AFWNVVNWADVAK) were detected. Comparisons were then made to all protein sequence in the NCBI database. The results of a BLAST search indicated that the internal sequences had high homology with a superoxide dismutase (SOD) proteins product from *T. fusca* YX (Accession number 289018.1). We named the protein as Tfu-lac.

### Dye oxidation properties

**Figure 6:**
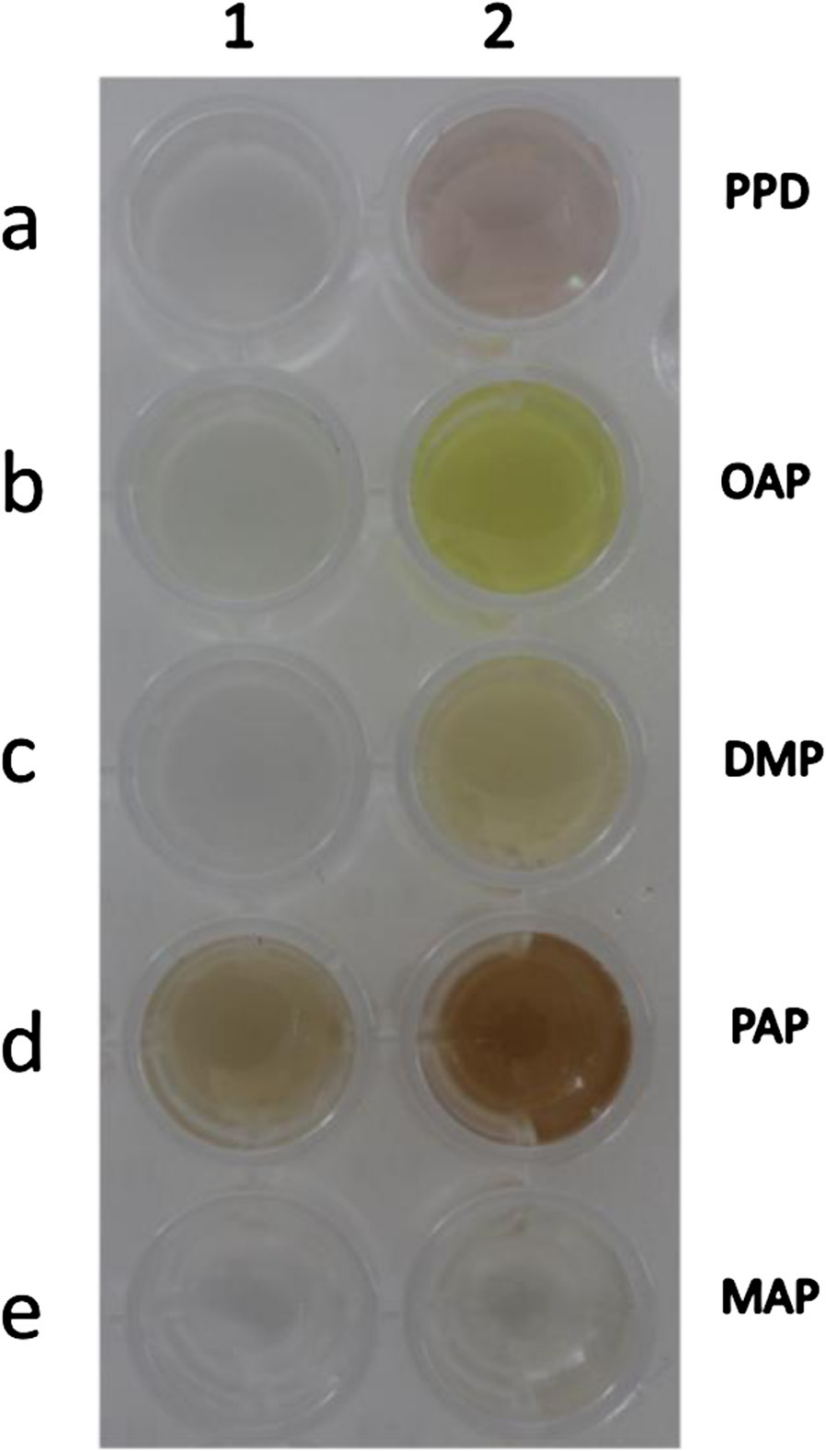
**The result of the chromogenic reaction of laccase from *T. fusca *BCRC 19214 for the dye intermediates used in hair color.** Lane 1, control experiments were carried out without laccase; Lane 2, **(a)** PPD, *p*-phenylenediamine, **(b)** OAP, *o*-aminophenol, **(c)** DMP, 2,6-dimethylphenylalanine, **(d)** PAP, *p*-aminophenol, and **(e)** MAP *m*-aminophenol.

As shown in Table 5, the laccase showed oxidation activity for 5 dye intermediates, including *p*-phenylenediamine, 2,6-dimezthylphenylalanine, *o*-aminophenol, *p*-aminophenol and *m*-aminophenol at pH 8.0. The chromogenic reactions of laccase for the dye oxidation widely used in hair color are shown in Figure 6. The laccase exhibited the higher activities toward 2,6-dimethylphenylalanine and *p*-aminophenol. In contrast, the oxidation effect of laccase was less for *m*-aminophenol.

**Table 5:** **Oxidation activities of laccase from *T. fusca *BCRC 19214 for dye intermediates**

**Substrate**	**Relative activity (%)**			
	**Tfu**	Flac1^*^	BOD^*^	RvL^*^
*p*-phenylenediamine	47.3	800.0	29.4	47.4
*o*-aminophenol	71.6	**2,900.0**	**547.0**	**274.7**
2,6-dimethylphenylalanine	**100.0**	100.0	100.0	100.0
*p*-aminophenol	96.0	100.0	11.7	9.0
*m*-aminophenol	0.4	-	-	-

*Data from Saito et al., 2012.

## Discussion

Thermophilic actinomycetes are of particular interest because they produce a variety of thermostable enzymes that are involved in the degradation process (Tuncer and Ball, 2002; Yang and Liu, 2004; Yang et al., 2007; Yang and Liu, 2008; Yang et al., 2010; Huang et al. 2010; Huang et al., 2011). *T. fusca* seems to be unique among thermophilic actinomycetes in having a laccase.

Some laccases had been isolated from microorganisms and the properties were summarized in Table 6. Compared with other laccases, the optimum temperature and optimum pH varied from 40°C to 85°C and 3.0 to 9.0, respectively. The laccases from *Bacillus subtilis* and *Pseudomonas aerophilum* had the highest optimal temperature. However, the optimal pH of these two enzymes retained in neutral to acidic (Martins et al., 2002; Fernandes et al., 2010). The laccase from *Streptomyces sviceus* had the wild pH range (5.0-11.0). Approximately 50% of the original activity remained after treatment at 50°C for 226 min. (Gunne and Urlacher, 2012). The laccase purified from *T. fusca* BCRC 19214 had the similar wild pH range with the property from *S. sviceus.* The thermostability of the laccase from *T. fusca* BCRC 19214 was better than the laccase from *S. sviceus.* Therefore, the laccase purified from *T. fusca* BCRC 19214 was an excellent thermo-alkali-stable laccase for hair coloring and further industrial applications.

**Table 6:** Biochemical properties of some purified laccase

**Source**	**Optimum temperature**	**Optimum pH**	**MW (kDa)**	**Reference**
*Trametes versicolor*	40	4.0-5.0	66	Minussi et al., 2007
*Ganoderma lucidum*	60	3	68	Ding et al., 2012
*Botrytis cinerea*	60	3.5	74	Slomczynski et al., 1995
*Chaetomium thermophile*	60	6	77	Chefetz et al., 1998
*Cladosporium cladosporioides*	50	3.5	75.17	Vijaykumar et al., 2011
*Schyzophylum commune*	40	6	63	Irshad et al., 2011
*Pycnoporus cinnabarinus*	-	4	81	Eggert et al., 1996
*Flammulina velutipes*	-	5	28	Saito et al., 2012
*Bacillus subtilis*	85	4.2	65	Martins et al., 2002
*Pseudomonas aerophilum*	85	3	49.6	Fernandes et al., 2010
*Streptomyces sviceus*	-	9	32.5	Gunne and Urlacher 2012
*Streptomyces psammoticus*	45	8.5	43	Niladevi et al., 2008
*Paraconiothyrium variabile*	50	4.8	84	Forootanfar et al., 2011

Addition of 10 mM SDS lead to a reduction of laccase activity to 63%. Addition of 10 mM sodium azide, a well-known laccase inhibitor, led to a decrease of activity by 20%, whereas many laccases are completely inhibited by concentrations in the micromolar range (Gunne and Urlacher, 2012). The relative stability of the laccase from *T. fusca* BCRC 19214 with chemicals allows use of the enzyme in a wide variety of reaction compositions.

The laccse activity was reduced by metal ions of Hg. It was similar with the results from *Strpeomyces psammoticus* (Niladevi et al., 2008), *Trametes hirsute* (Couto and Herrera, 2006), and *Paraconiothyrium variabile* (Forootanfar et al., 2011). The role of copper in the enhancement of laccase activity has been well demonstrated in both fungi and bacteria (Givaudan et al., 1993). Similar result has been reported from *Streptomyces cyaneus* (Arias et al., 2003). However, the present result on the effect of Cu on the purified laccase was no enhancement effects.

With 20% water miscible solvent like acetonitrile and DMF in the reaction system, the laccase increased the activities to 110-118%. They were suggested to weaken the hydrophobic interaction and increased the stability of laccase in aqueous solutions (Kovrigin and Potekhin, 2000). With 40% solvent like DMSO, methanol, 2-propanol, acetonitrile, and aceton, the laccase activity from *Streptomyces sviceus* dropped to 20-40% (Gunne and Urlacher, 2012). The effects of organic solvent on tha activity of purified laccase from *T. fusca* BCRC 19214 were similar with the results from *S. sviceus*.

This enzyme differed from the sequence of the laccase-like 24.7-kDa copper-containing oxidase Tfu1114 (Genebank number AAZ55152.1) secreted from *T. fusca* (Chen et al., 2013). The results of a BLAST search also indicated that the deduced amino acid sequence of the Tfu-lac protein had higher homologies with five superoxide dismutase (SOD) proteins (YP_007685998.1, YP_062104.1, YP_006642575.1, YP_003678569.1, YP_001625417.1).

Saito et al. investigated the oxidation activities of laccase Flac1 from *Flammulina velutipes* (Saito et al., 2012), bilirubin oxidase (BOD) from *Myrothecium verrucaria* (Guo et al., 1991), and laccase (RvL) from *Rhus vernicifera* (Sulistyaningdyah et al., 2004). All of these enzymes showed the highest activity toward *o*-aminophenol, which differ from the laccase in this study. The laccase from *T. fusca* displayed better activity toward 2,6-dimethylphenylalanine and *p*-aminophenol. This property could be used for developing new dye colors from the intermediates oxidation process.

## Competing interests

The authors declare that they have no competing interests.

## References

[B1] Albino A Dias AA Bezerra RM Pereira AN Activity and elution profile of laccase during biological decolorization and dephenolization of olive mill wastewater Bioresour Technol 2004 3 7 13 10.1016/j.biortech.2003.08.006 14643980

[B2] Ames BN Kammen HO Yamasaki E Dyes are mutagenic: identification of a variety of mutagenic ingredients Proc Nat Acad Sci 1975 3 2423 2427 10.1073/pnas.72.6.2423 1094469 PMC432771

[B3] Arias ME Arenas M Rodriguez J Solveri J Ball AS Hernandez M Kraft pulp biobleaching and mediated oxidation of a non-phenolic substrate by laccase from *Streptomyces cyaneus* CECT3335 Appl Environ Microbiol 2003 3 1953 1958 10.1128/AEM.69.4.1953-1958.2003 12676669 PMC154780

[B4] Baldrian P Fungal laccases - occurrence and properties FEMS Microbiol Rev 2006 3 215 242 10.1111/j.1574-4976.2005.00010.x 16472305

[B5] Chefetz B Chen Y Hadar Y Purification and characterization of laccase from *Chaetomium thermophilium* and its role in humification Appl Environ Microbiol 1998 3 3175 3179 9726856 10.1128/aem.64.9.3175-3179.1998 PMC106706

[B6] Chen CY Hsieh ZS Cheepudom J Yang CH Meng M A 24.7-kDa copper-containing oxidase, secreted by *Thermobifida fusca*, significantly increasing the xylanase/cellulase-catalyzed hydrolysis of sugarcane bagasse Appl Microbiol Biotechnol 2013 10.1007/s00253-013-4727-y 23377789

[B7] Couto SR Herrera JLT Industrial and biotechnological applications of laccases: A review Biotechnol Adv 2006 3 500 513 10.1016/j.biotechadv.2006.04.003 16716556

[B8] Ding Z Peng L Chen Y Zhang L Gu Z Shi G Zhang K Production and characterization of thermostable laccase from the mushroom, *Ganoderma lucidum*, using submerged fermentation Afr J Microbiol Res 2012 3 1147 1157

[B9] Eggert C Temp U Eriksson KEL The ligninolytic system of the white rot fungus *Pycnoporus cinnabarinus*: purification and characterization of the laccase Appl Environ Microbiol 1996 3 1151 1158 8919775 10.1128/aem.62.4.1151-1158.1996 PMC167880

[B10] Fernandes AT Damas JM Todorovic S Huber R Baratto MC Pogni R Soares CM Martins LO The multicopper oxidase from the archaeon *Pyrobaculum aerophilum* shows nitrous oxide reductase Activity FEBS J 2010 3 3176 3189 10.1111/j.1742-4658.2010.07725.x 20597980

[B11] Forootanfar H Faramarzi MA Shahverdi AR Yazdi MT Purification and biochemical characterization of extracellular laccase from the ascomycete *Paraconiothyrium variabile* Bioresour Technol 2011 3 1808 1814 10.1016/j.biortech.2010.09.043 20933400

[B12] Giardina P Faraco V Pezzella C Piscitelli A Vanhulle S Sannia G Laccases: a never-ending story Cell Mol Life Sci 2010 3 369 385 10.1007/s00018-009-0169-1 19844659 PMC11115910

[B13] Givaudan A Effose A Faure D Potier P Bouillant ML Bally R Polyphenol oxidase from *Azospirillum lipoferum* isolated from rice rhizosphere: evidence for laccase activity in non-motile strains of *Azospirillum lipoferum* FEMS Microbiol Lett 1993 3 205 210 10.1111/j.1574-6968.1993.tb06100.x

[B14] Gouka RJ van der Heiden M Swarthoff T Verrips CT Cloning of a phenol oxidase gene from *Acremonium murorum* and its expression in *Aspergillus awamori* Appl Environ Microbiol 2001 3 2610 2616 10.1128/AEM.67.6.2610-2616.2001 11375170 PMC92914

[B15] Gunne M Urlacher VB Characterization of the alkaline laccase Ssl1 from *Streptomyces sviceus* with unusual properties discovered by genome mining PLoS One 2012 3 1 8 10.1371/journal.pone.0052360 PMC3527528 23285009

[B16] Guo J Liang XX Mo PS Li GX Purification and properties of bilirubin oxidase from *Myrothecium verrucaria* Appl Biochem Biotechnol 1991 3 135 143 10.1007/BF02921784 1799289

[B17] Heinzkill M Bech L Halkier T Schneider P Anke T Characterization of laccases and peroxidases from wood-rotting fungi (family *Coprinaceae*). Appl Environ Microbiol 1998 3 1601 1606 10.1128/aem.64.5.1601-1606.1998 PMC106202 9572923

[B18] Huang YC Chen GH Chen YF Chen WL Yang CH Heterologous expression of thermostable acetylxylan esterase gene from *Thermobifida fusca* and its synergistic action with xylanase for the production of xylooligosaccharides Biochem Biophys Res Commun 2010 3 718 723 10.1016/j.bbrc.2010.08.136 20816933

[B19] Huang YC Chen YF Chen CY Chen WL Ciou YP Liu WH Yang CH Production of ferulic acid from lignocellulolytic agricultural biomass by *Thermobifida fusca* thermostable esterase produced in *Yarrowia lipolytica* transformant Bioresour Technol 2011 3 8117 8122 10.1016/j.biortech.2011.05.062 21683590

[B20] Irshad M Asgher M Sheikh MA Nawaz H Purification and characterization of laccase produced by *Schyzophylum commune* IBL-06 IN solid state culture of banana stalks BioResources 2011 3 2861 2873

[B21] Kovrigin EL Potekhin SA On the stabilizing action of protein denaturants: acetonitrile effect on stability of lysozyme in aqueous solutions Biophys Chem 2000 3 45 59 10.1016/S0301-4622(99)00122-2 10631479

[B22] Kües U Rühl M Multiple multi-copper oxidase gene families in basidiomycetes - what for? Curr Genom 2011 3 72 94 10.2174/138920211795564377 PMC3129051 21966246

[B23] Locci R Williams ST, Sharpe E, Holt JG Streptomycetes and related genera Bergey’s manual of systematic bacteriology, vol 4 1989 Baltimore: Williams and Wilkins 2451 2508

[B24] Lykidis A Mavromatis K Ivanova N Anderson I Land M DiBartolo G Martinez M Lapidus A Lucas S Copeland A Richardson P Wilson DB Kyrpides N Genome sequence and analysis of the soil cellulolytic actinomycete *Thermobifida fusca* YX J Bacteriol 2007 3 2477 2486 10.1128/JB.01899-06 17209016 PMC1899369

[B25] Martins LO Soares CM Pereira MM Teixeira M Costa T Jones GH Henriques AO Molecular and biochemical characterization of a highly stable bacterial laccase that occurs as a structural component of the *Bacillus subtilis* endospore coat J Biol Chem 2002 3 18849 18859 10.1074/jbc.M200827200 11884407

[B26] Minussi RC Miranda MA Silva JA Ferreira CV Aoyama H Marangoni S Rotilio D Pastore GM Durán N Purification, characterization and application of laccase from *Trametes versicolor* for colour and phenolic removal of olive mill wastewater in the presence of 1-hydroxybenzotriazole Afr J Biotechnol 2007 3 1248 1254

[B27] Mukhopadhyay A Dasgupta AK Chakrabarti K Thermostability, pH stability and dye degrading activity of a bacterial laccase are enhanced in the presence of Cu_2_O nanoparticles Bioresour Technol 2013 3 25 36 23131620 10.1016/j.biortech.2012.09.087

[B28] Niladevi KN Jacob N Prema P Evidence for a halotolerant-alkaline laccase in *Streptomyces psammoticus*: purification and characterization Process Biochem 2008 3 654 660 10.1016/j.procbio.2008.02.002

[B29] Saito K Ikeda R Endo K Tsujino Y Takagi M Tamiya E Isolation of a novel alkaline-induced laccase from *Flammulina velutipes* and its application for hair coloring J Biosci Bioeng 2012 3 575 579 10.1016/j.jbiosc.2012.01.001 22300716

[B30] Slomczynski D Nakas JP Tanenbaum SW Production and characterization of laccase from *Botrytis cinerea* 61*–*34 Appl Environ Microbiol 1995 3 907 912 16534974 10.1128/aem.61.3.907-912.1995 PMC1388373

[B31] Sulistyaningdyah WT Ogawa J Tanaka H Maeda C Shimizu S Characterization of alkaliphilic laccase activity in the culture supernatant of *Myrothecium verrucaria* 24G-4 in comparison with bilirubin oxidase FEMS Microbiol Lett 2004 3 209 214 10.1016/S0378-1097(03)00892-9 14757242

[B32] Tuncer M Ball AS Degradation of lignocellulose by extracellular lignocellulolytic enzyme produced by *Thermomonospora fusca* BD25 Appl Microbiol Biotechnol 2002 3 608 611 10.1007/s00253-001-0894-3 11956742

[B33] Vijaykumar M Halaburgi VM Sharma S Sinha M Singh TP Karegoudar TB Purification and characterization of a thermostable laccase from the ascomycetes *Cladosporium cladosporioides* and its applications Process Biochem 2011 3 1146 1152 10.1016/j.procbio.2011.02.002

[B34] Yang CH Huang YC Chen CY Degradation of rutin by *Thermoactinomyces vulgaris* and other thermophilic compost isolates J Agric Food Chem 2009 3 5095 5099 10.1021/jf900617z 19489631

[B35] Yang CH Huang YC Chen CY Wen CY Heterologous expression of *Thermobifida fusca* thermostable α-amylase in *Yarrowia lipolytica* and its application in boiling stable resistant sago starch preparation J Ind Microbiol Biot 2010 3 953 960 10.1007/s10295-010-0745-2 20495998

[B36] Yang CH Liu WH Purification and properties of a maltotruose-producing α-amylase from *Thermobifida fusca* Enzyme Microb Technol 2004 3 254 260 10.1016/j.enzmictec.2004.05.004 22578869

[B37] Yang CH Liu WH Purification and properties of an acetylxylan esterase from *Thermobifida fusca* Enzyme Microb Technol 2008 3 181 186 10.1016/j.enzmictec.2007.09.007 22578869

[B38] Yang CH Yang SF Liu WH Production of xylooligosaccharides from xylans by extracellular xylanases from *Thermobifida fusca* J Agric Food Chem 2007 3 3955 3959 10.1021/jf0635964 17432873

